# 

**Published:** 1874-09

**Authors:** 


Vol. XIV] SEPTEMBER, 1874. [No. 2.

### BUFFALO

### Medical and Surgical Journal.

EDITED BY JULIUS F. MINER, M. D.

Professor of Special Surgery in the Buffalo Medical College ; Surgeon to the Buffalo General Hospital ; Surgeon to Buffalo Hospital of the Sisters of Charity, etc., etc.

AND

EDWARD N. BRUSH, M. D.

BUFFALO.

PUBLISHED FOR THE EDITORS.

1874.

